# Supplementary material for: Two-step chromosome segregation in the stalked budding bacterium Hyphomonas neptunium
Source: Nat Commun. 2019 Jul 23;10:3290. doi: 10.1038/s41467-019-11242-5 (PMC6650430; doi:10.1038/s41467-019-11242-5)
Supplement: Supplementary file 1 — Supplementary Information [file 41467_2019_11242_MOESM1_ESM.pdf]

Supplementary material

Two-step chromosome segregation in the stalked budding bacterium *Hyphomonas neptunium*

Jung *et al.*

Supplementary figures

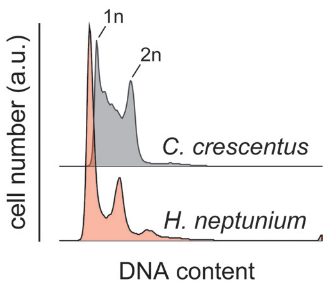

Supplementary Figure 1. DNA content of *H. neptunium*. *H. neptunium* ATCC 15444 and *Caulobacter crescentus* CB15N were grown in MB and PYE medium, respectively, and treated for 25 min with Vybrant DyeCycle Orange to stain the chromosomal DNA. Subsequently, the cellular DNA content was measured by flow cytometry (n=30.000 cells each). Note that the chromosomes of *H. neptunium* (~3.7 Mbp) and *C. crescentus* (~4.0 Mbp) have approximately the same size. The peaks corresponding to one (1n) and two (2n) chromosome equivalents are indicated.

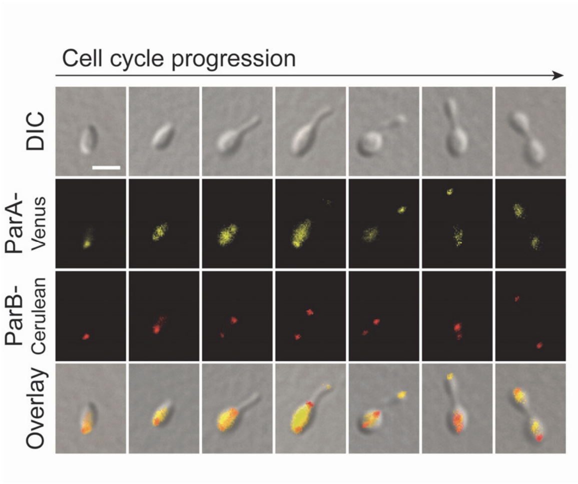

Supplementary Figure 2. Localization of ParA and ParB at different stages of the cell cycle. Strain AJ80 (*parB-cerulean* Pcu::Pcu-*parA-venus*) was grown in MB medium, induced for 4 h with 0.3 mM $CuSO_4$, and analyzed by DIC and fluorescence microscopy. Shown are representative images of cells at different stages of the developmental cycle. Bar: 1.5 µm.

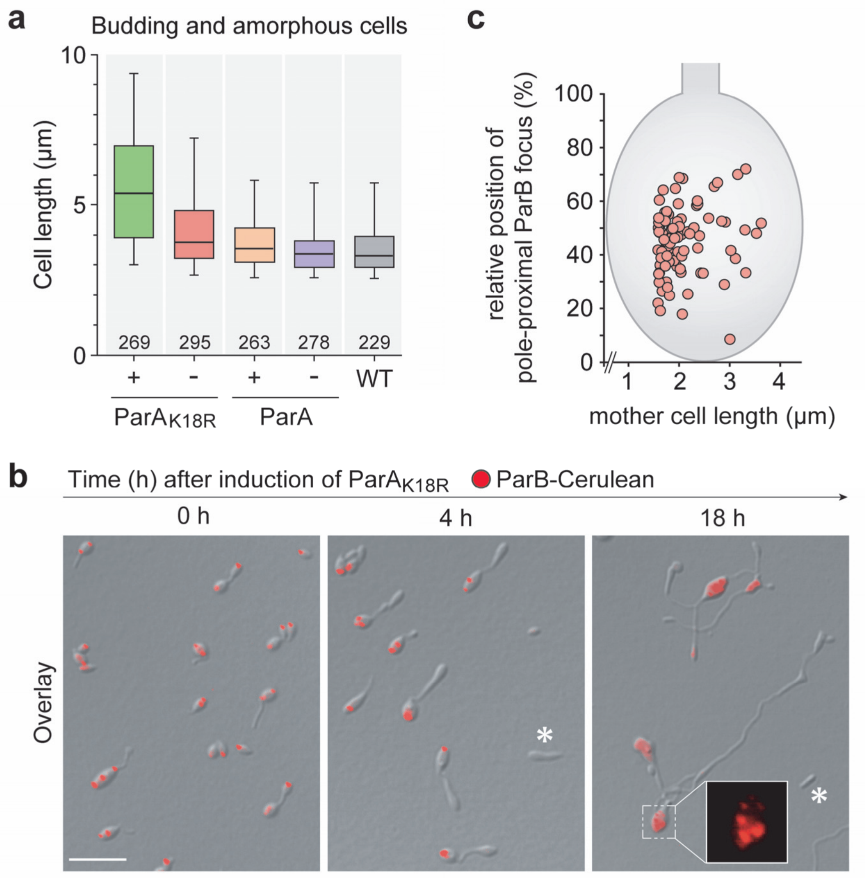

Supplementary Figure 3. Inhibition of *ori* segregation upon induction of a dominant negative ParA variant. **(a)** Distribution of cells lengths in the cultures analyzed in Figure 5c. The data are represented as box plots. The horizontal line indicates the median, the box the interquartile range, and the whiskers the 5th and 95th percentiles. The number of cells analyzed is given underneath the boxes. Source data are provided as a Source Data file. **(b)** Phenotype of cells producing the dominant negative ParA variant ParA$_{K18R}$-Venus. Strain AJ79 (*parB-cerulean* P_{Cu}::P_{Cu}-*parA$_{K18R}$-venus*) was pre-grown in MB medium, induced with 0.5 mM $CuSO_4$, and cultivated for another 18.5 h. Samples were taken at the indicated time points and subjected to microscopy. Shown are overlays of DIC images and the corresponding ParB-Cerulean signals. Asterisks indicate cells without a ParB-Cerulean signal. Bar 5 µm. **(c)** Localization of ParB in the cells described in (b) after induction of ParA$_{K18R}$-Venus for 4 h. The graph shows the relative subcellular position of the ParB-Cerulean focus that was closest to the stalk base plotted versus the total length of the mother cell body (n=91 cells), with 0% indicating the stalk-distal pole and 100% the stalked pole.

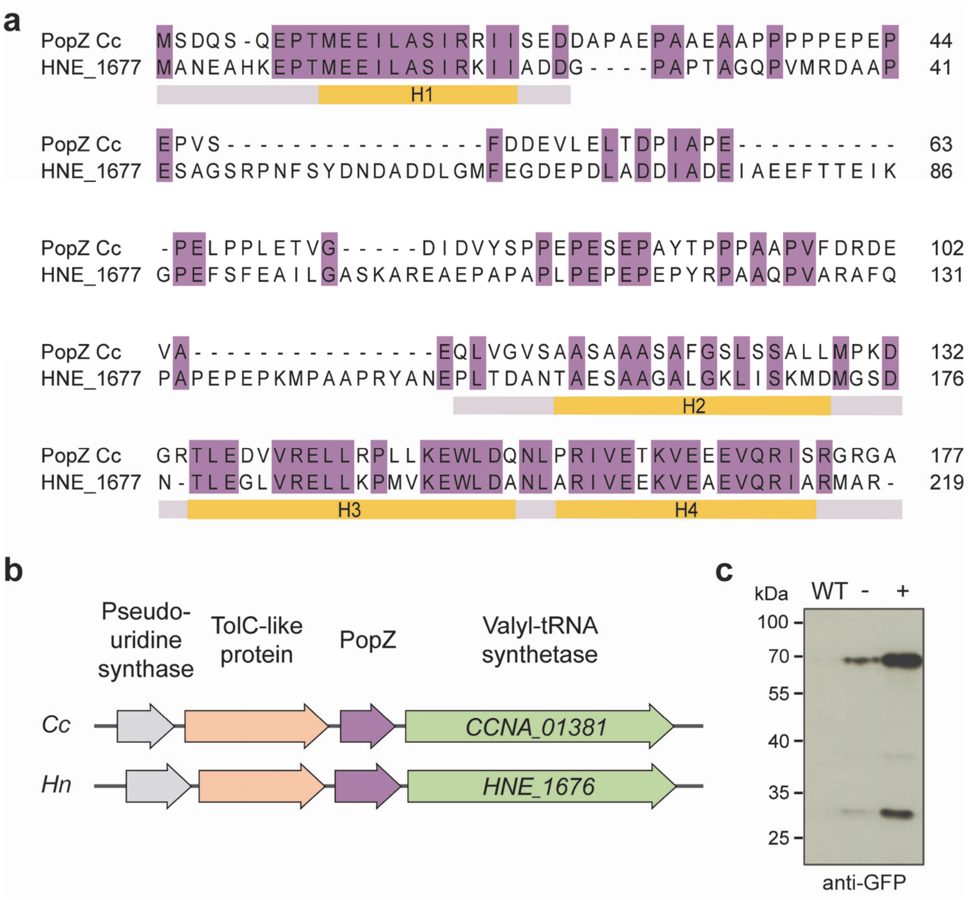

Supplementary Figure 4. Analysis of the *H. neptunium* PopZ homolog. **(a)** Alignment of the predicted amino acid sequences of PopZ from *C. crescentus* (*Cc*; CCNA_01380) and *H. neptunium* (*Hn*; HNE_1677). Conserved residues are highlighted in purple. Light grey bars indicate the N- and C-terminal regions previously shown to be critical for function[1,2]. Orange bars denote predicted α-helices. **(b)** Chromosomal context of *popZ* in *C. crescentus* and *H. neptunium*. Homologous genes are shown in the same color. **(c)** Immunoblot showing the induction of PopZ-Venus. Strain AJ34 (P_{Zn}::P_{Zn}-*popZ-venus*) was grown in MB medium and induced for 23 h with 0.5 mM $ZnSO_4$. Before (-) and after induction (+), samples were taken and subjected to immunoblot analysis with anti-GFP antibodies. Wild-type cells (WT) grown in MB medium were analyzed as a control. Similar to its *C. crescentus* homolog[1,2], PopZ from *H. neptunium* migrates more slowly than expected in SDS gels (predicted molecular weight: 54 kDa).

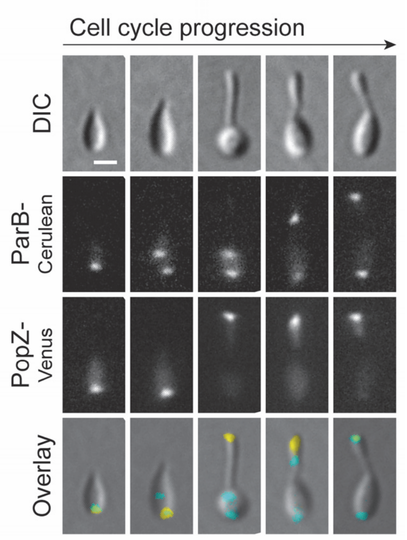

Supplementary Figure 5. Colocalization of PopZ with ParB during the early and late stages of the cell cycle. Strain AJ90 (*parB-cerulean* P_{Zn}::P_{Zn}-*popZ-venus*) was induced for 2.5 h with 0.3 mM $ZnSO_4$ and analyzed by DIC and fluorescence microscopy. Shown are representative cells at different stages of the cell cycle. Bar: 1 µm.

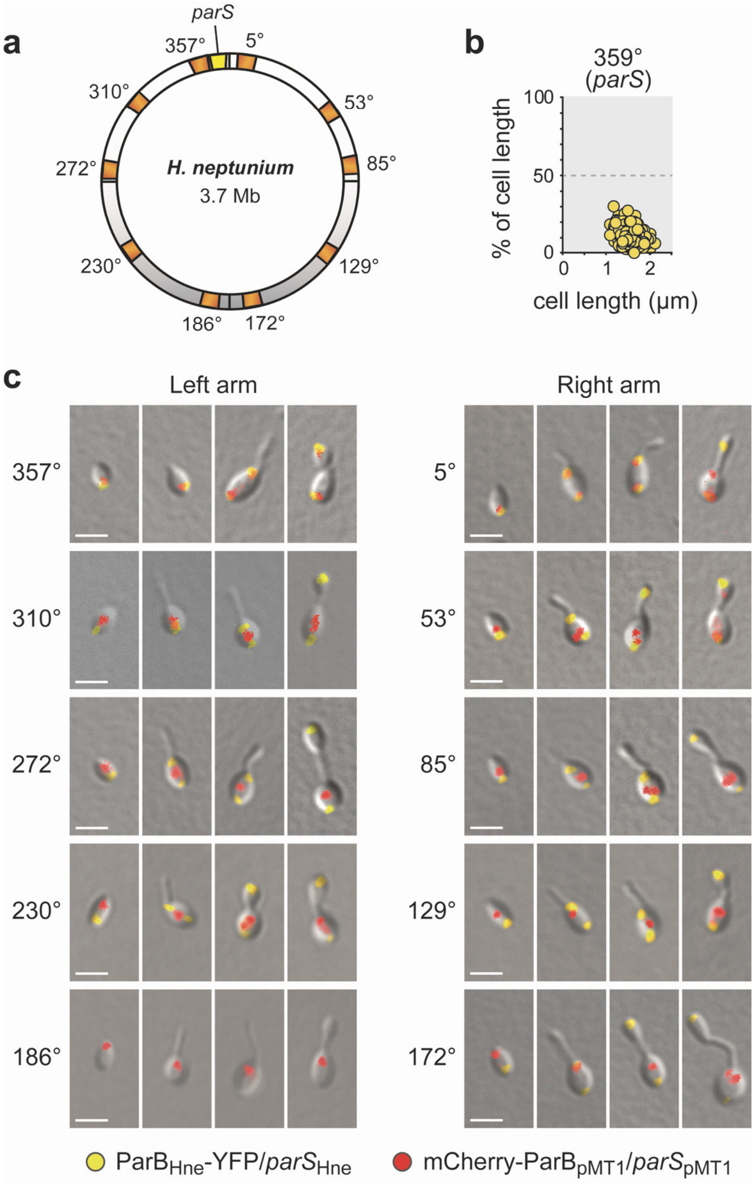

Supplementary Figure 6. Organization and dynamics of the *H. neptunium* chromosome. **(a)** Schematic showing the locations chromosomal loci tagged with the *Y. pestis* ParB-*parS*$_{pMT1}$ system. **(b)** Localization of the ParB·*parS* complex in G1-phase. Strain KH22 (*parB-yfp*) was grown in MB medium and analyzed by DIC and fluorescence microscopy. The relative subcellular position of ParB-YFP was determined in swarmer and early stalked cells and plotted versus the total cell length (n=315), with 0% indicating the old (flagellated) pole and 100% the future stalked pole. **(c)** Subcellular localization of ten different chromosomal loci distributed evenly across the *H. neptunium* chromosome. Strains (*parB-yfp* P$_{Zn}$::P$_{Zn}$-*mCherry-parB*$_{pMT1}$) carrying *parS*$_{pMT1}$ at the indicated chromosomal locations (AJ64-69, SRE13-15, AJ49) were grown in MB medium and analyzed by DIC and fluorescence microscopy. Shown are representative snap-shot images of cells at different stages of their cell cycle. Note that strain AJ49 (186°) does not carry a *parB-yfp* fusion and thus only shows the mCherry-ParB$_{pMT1}$ signal. Bar: 1.5 μm.

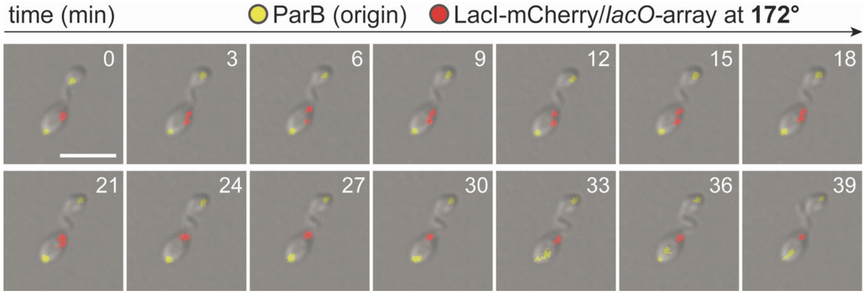

Supplementary Figure 7. Segregation of the chromosomal *ter* region. Cells of strain AJ86 (*HNE_1729*::*lacO*$_n$ *parB-yfp* P_{Zn}::P_{Zn}-*lacI-mCherry*) were induced for 3 h with 0.3 mM $ZnSO_4$, transferred to an MB-agarose pad, and imaged at 3 min intervals. Shown are overlays of DIC and fluorescence images. Bars: 3 µm.

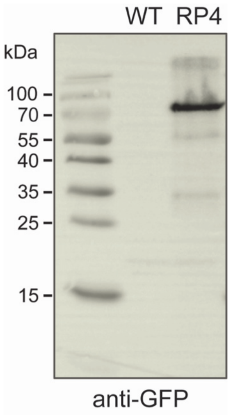

Supplementary Figure 8. Stability of the DnaN-Venus fusion. Cells of strains ATCC 15444 (WT) and RP4 (*dnaN-venus*) were grown in MB medium and subjected to immunoblot analysis with anti-GFP antibodies.

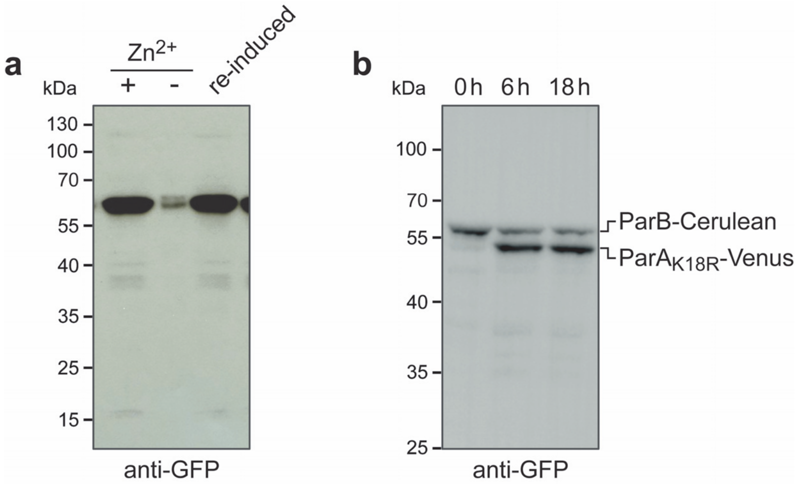

Supplementary Figure 9. Full scans of the immunoblots shown in Figures 4 and 5. (A) Full scan of the of the immunoblot shown in Figure 4A. Strain AJ46 (Δ*parA* P_{Zn}::P_{Zn}-*parA-venus*) was grown in MB medium containing inducer (0.3 mM $ZnSO_4$), washed, shifted to $ZnSO_4$-free medium, and cultivated for another 45 h to deplete ParA-Venus. Subsequently, cells were transferred to MB medium contianing 0.5 mM $ZnSO_4$ and cultivated for 24 h to re-induce the synthesis of the fusion protein. Samples were subjected to immunoblot analysis with anti-GFP antibodies. **(B)** Full scan of the immunoblot shown in Figure 5B. Shown are the levels of $ParA_{K18R}$-Venus and ParB-Cerulean in strain AJ79 (*parB-cerulean* P_{Cu}::P_{Cu}-*parA*$_{K18R}$-*venus*) grown in the absence or presence of inducer. Cells were pre-grown in MB medium, induced with 0.5 mM $CuSO_4$, and cultivated for another 18.5 h. Samples were taken at the indicated time points and subjected to immunoblot analysis with anti-GFP antibodies.

Supplementary tables

Supplementary Table 1. Strains used in this study.

Strain	Genotype	Construction/Reference
***E. coli* strains**		
TOP10	cloning strain	Invitrogen
WM3064	*thrB*1004 *pro thi rpsL hsdS lacZ*ΔM15 RP4–1360 Δ(*araBAD)567* Δ*dapA*1341::[*erm pir*(wt)]	W. Metcalf (unpublished)
Rosetta(DE3) pLysS	F^- *ompT hsdS*$_B$(r_{B-} m_{B-}) *gal dcm* (DE3) pLysSRARE (Cam^R)	Merck Millipore
***H. neptunium* strains**		
ATCC 15444	wild type (*aka* LE670)	[3]
AJ34	P_{Zn}::P_{Zn}-*HNE_1677 (popZ)-venus*	Transformation of ATCC 15444 with pAJ34
AJ38	Δ*HNE_1677 (popZ)*	Deletion of *HNE_1677* in ATCC 15444 using pAJ38
AJ46	Δ*parA* P_{Zn}::P_{Zn}-*parA-venus*	Deletion of *parA* in AJ73 using pAJ46
AJ49	P_{Zn}::P_{Zn}-*mCherry-parB*$_{pMT1}$ *parS*$_{pMT1}$ at 186°	Transformation of AR58 with pAJ36
AJ57	*parB-yfp parS*$_{pMT1}$ at 172°	Transformation of KH22 with pAJ62
AJ58	*parB-yfp parS*$_{pMT1}$ at 272°	Transformation of KH22 with pAJ63
AJ59	*parB-yfp parS*$_{pMT1}$ at 85°	Transformation of KH22 with pAJ64
AJ60	*parB-yfp parS*$_{pMT1}$ at 53°	Transformation of KH22 with pAJ65
AJ61	*parB-yfp parS*$_{pMT1}$ at 357°	Transformation of KH22 with pAJ66
AJ62	*parB-yfp parS*$_{pMT1}$ at 5°	Transformation of KH22 with pAJ67
AJ64	*parB-yfp* P_{Zn}::P_{Zn}-*mCherry-parB*$_{pMT1}$ *parS*$_{pMT1}$ at 172°	Transformation of AJ57 with pAJ70
AJ65	*parB-yfp* P_{Zn}::P_{Zn}-*mCherry-parB*$_{pMT1}$ *parS*$_{pMT1}$ at 272°	Transformation of AJ58 with pAJ70
AJ66	*parB-yfp* P_{Zn}::P_{Zn}-*mCherry-parB*$_{pMT1}$ *parS*$_{pMT1}$ at 85°	Transformation of AJ59 with pAJ70
AJ67	*parB-yfp* P_{Zn}::P_{Zn}-*mCherry-parB*$_{pMT1}$ *parS*$_{pMT1}$ at 53°	Transformation of AJ60 with pAJ70
AJ68	*parB-yfp* P_{Zn}::P_{Zn}-*mCherry-parB*$_{pMT1}$ *parS*$_{pMT1}$ at 357°	Transformation of AJ61 with pAJ70
AJ69	*parB-yfp* P_{Zn}::P_{Zn}-*mCherry-parB*$_{pMT1}$ *parS*$_{pMT1}$ at 5°	Transformation of AJ62 with pAJ70
AJ73	P_{Zn}::P_{Zn}-*parA-venus*	Transformation of ATCC 15444 with pAJ45
AJ76	*parB-cerulean*	Replacement of *parB* with *parB-cerulean* in ATCC 15444 using pAJ74
AJ79	*parB-cerulean* P_{Cu}::P_{Cu}-*parA(K18R)-venus*	Transformation of AJ76 with pAJ75
AJ80	*parB-cerulean* P_{cu}::P_{cu}-*parA-venus*	Transformation of AJ76 with pAJ78
AJ86	*HNE_1729*::lac*O*$_n$ *parB-yfp* P_{Zn}::P_{Zn}-*lacI-mCherry*	Transformation of KH23 with pAJ79
AJ87	*HNE_3540*::lac*O*$_n$ *parB-yfp* P_{Zn}::P_{Zn}-*lacI-mCherry*	Transformation of KH23 with pAJ80
AJ89	Δ*HNE_1677* (*popZ*) *parB*::*parB-yfp*	Transformation of AJ38 with pSW55
AJ90	*parB-cerulean* P_{Zn}::P_{Zn}-*popZ-venus*	Transformation of AJ76 with pAJ34
RP4	*dnaN-venus*	Replacement of *dnaN* with *dnaN-venus* in ATCC 15444 using pAJ84
JR47	*parB-cerulean dnaN-Venus*	Replacement of *dnaN* with *dnaN-venus* in AJ76 using pAJ84
JR50	*HNE_0032*::lac*O*$_n$ *parB-yfp* P_{Zn}::P_{Zn}-*lacI-mCherry*	Transformation of KH23 with pJR82
AR48	*parB*::*parB-yfp*	Transformation of ATCC 15444 with pSW55
AR58	P_{Zn}::P_{Zn}-*mCherry-parB*$_{pMT1}$	Transformation of ATCC 15444 with pAR51
KH14	P_{Zn}::P_{Zn}-*lacI-mCherry*	Transformation of ATCC 15444 with pKH9
KH22	*parB-yfp*	Replacement of *parB* with *parB-yfp* in ATCC 15444 using pKH15
KH23	*parB-yfp* P_{Zn}::P_{Zn}-*lacI-mCherry*	Replacement of *parB* with *parB-yfp* in KH14 using pKH15
KH24	*gyrB*::lac*O*$_n$, *parB-yfp* P_{Zn}::P_{Zn}-*lacI-mCherry*	Transformation of KH23 with pKH4
SRE5	*parB-yfp parS*$_{pMT1}$ at 129°	Transformation of KH22 with pSRE8
SRE6	*parB-yfp parS*$_{pMT1}$ at 230°	Transformation of KH22 with pSRE9
SRE7	*parB-yfp parS*$_{pMT1}$ at 310°	Transformation of KH22 with pSRE10
SRE13	*parB-yfp* P_{Zn}::P_{Zn}-*mCherry-parB*$_{pMT1}$ *parS*$_{pMT1}$ at 129°	Transformation of SRE5 with pSRE16
SRE14	*parB-yfp* P_{Zn}::P_{Zn}-*mCherry-parB*$_{pMT1}$ *parS*$_{pMT1}$ at 230°	Transformation of SRE6 with pSRE16
SRE15	*parB-yfp* P_{Zn}::P_{Zn}-*mCherry-parB*$_{pMT1}$ *parS*$_{pMT1}$ at 310°	Transformation of SRE7 with pSRE16

Supplementary Table 2. Plasmids used in this study.

Plasmid	Description	Reference
**Basic vectors**		
pCCFPN-3	Integrating plasmid for generation of N-terminal CFP fusions under control of P_{Cu}, Rif^R	[4]
pCERC-2	Integrating plasmid for generation of C-terminal Cerulean fusion at site of interest, Kan^R	[5]
pCVENC-2	Integrating plasmid for generation of C-terminal Venus fusion under control of P_{Cu}, Kan^R	[4]
pCVENC-8	Integrating plasmid for generation of C-terminal Venus fusion under control of P_{Cu}, Tric^R	unpublished
pET21a+	Plasmid for overexpression of C-terminally His_6-tagged proteins, Amp^R	Novagen
pLAU43	Plasmid carrying a *lacO*$_n$ array, Kan^R	[6]
pLAU53	Plasmid carrying P_{BAD}-*lacI-ecfp tetR-eyfp*, Amp^R	[6]
pMCS-2	Integrating plasmid containing multiple cloning site, Kan^R	[5]
pMCS-3	Integrating plasmid containing multiple cloning site, Rif^R	[5]
pMS138	Plasmid carrying *parS*$_{pMT1}$, Gent^R	[7]
pMS226	Plasmid carrying *mCherry-parB*$_{pMT1}$ under the control of P_{van}, Kan^R	[7]
pNPTS138	*sacB*-containing suicide vector used for double homologous recombination, Kan^R	M. R. K. Alley, unpublished
pXCHYC-3	Integrating plasmid for generation of C-terminal mCherry fusions under control of P_{xyl}, Rif^R	[5]
pYFPC-2	Integrating plasmid for generation of C-terminal YFP fusions at site of interest, Kan^R	[5]
pZCHYN-3	Integrating plasmid for generation of N-terminal mCherry fusions under control of P_{Zn}, Rif^R	[4]
pZVENC-2	Integrating plasmid for generation of C-terminal Venus fusions under control of P_{Zn}, Kan^R	[4]
**Plasmids constructed in this work**		
pAJ34	pZVENC-2 carrying *popZ* (*HNE_1677*), Kan^R	This study
pAJ36	pAR46 (contains *parS*$_{pMT1}$) carrying part of *HNE_1854*, Rif^R	This study
pAJ38	pNPTS138 derivative for in-frame deletion in *popZ* (*HNE_1677*), Kan^R	This study
pAJ40	pET21a+ carrying *parB* (*HNE_3560*), Amp^R	This study
pAJ45	Integrating plasmid carrying *parA* (*HNE_3561*)-*venus* under the control of P_{Zn}, Rif^R	This study
pAJ46	pNPTS138 derivative for in-frame deletion in *parA* (*HNE_3561*), Kan^R	This study
pAJ54	pCERC-2 carrying *parB* (*HNE_3560*)	This study
pAJ56	pMCS-3 carrying *parS*$_{pMT1}$ and part of *HNE_1729*, Rif^R	This study
pAJ57	pMCS-3 carrying *parS*$_{pMT1}$ and part of *HNE_2644*, Rif^R	This study
pAJ58	pMCS-3 carrying *parS*$_{pMT1}$ and part of *HNE_0857*, Rif^R	This study
pAJ59	pMCS-3 carrying *parS*$_{pMT1}$ and part of *HNE_0550*, Rif^R	This study
pAJ60	pMCS-3 carrying *parS*$_{pMT1}$ and part of *HNE_3540*, Rif^R	This study
pAJ61	pMCS-3 carrying *parS*$_{pMT1}$ and part of *HNE_0063*, Rif^R	This study
pAJ62	pMCS-2 carrying *parS*$_{pMT1}$ and part of *HNE_1729*, Kan^R	This study
pAJ63	pMCS-2 carrying *parS*$_{pMT1}$ and part of *HNE_2644*, Kan^R	This study
pAJ64	pMCS-2 carrying *parS*$_{pMT1}$ and part of *HNE_0857*, Kan^R	This study
pAJ65	pMCS-2 carrying *parS*$_{pMT1}$ and part of *HNE_0550*, Kan^R	This study
pAJ66	pMCS-2 carrying *parS*$_{pMT1}$ and part of *HNE_3540*, Kan^R	This study
pAJ67	pMCS-2 carrying *parS*$_{pMT1}$ and part of *HNE_0063*, Kan^R	This study
pAJ70	pZCHYN-3 carrying *parB*$_{pMT1}$, Rif^R	This study
pAJ74	pNPTS138 derivative to replace *parB* with *parB-cerulean*, Kan^R	This study
pAJ75	pCVENC-2 carrying *parA* (*K18R*), Kan^R	This study
pAJ78	pCVENC-2 carrying *parA* (*HNE_3561*), Kan^R	This study
pAJ79	pKH3 carrying part of *HNE_1729*, Kan^R, Amp^R	This study
pAJ80	pKH3 carrying part of *HNE_3540,* Kan^R, Amp^R	This study
pAR25	Integrating plasmid carrying *parA* (*HNE_3561*)-*venus* under the control of P_{Zn}, Kan^R	This study
pAR46	pMCS-3 carrying *parS*$_{pMT1}$-sites, Rif^R	This study
pAR51	pZCHYN-2 carrying *parB*$_{pMT1}$, Kan^R	This study
pJR82	pKH3 carrying part of *HNE_0032*, Kan^R, Amp^R	
pKH3	pLAU43 carrying *oriT,* Kan^R, Amp^R	This study
pKH4	pKH3 carrying part of *gyrB,* Kan^R, Amp^R	This study
pKH9	Integrating plasmid carrying *lacI-mCherry* under the control of P_{Zn}, Rif^R	This study
pKH15	pNPTS138 derivative to replace *parB* with *parB-yfp*, Kan^R	This study
pSRE7	pCVENC-2 carrying *dnaN* , Kan^R	This study
pSRE8	pMCS-2 carrying *parS*$_{pMT1}$ and part of *HNE_1299*, Kan^R	This study
pSRE9	pMCS-2 carrying *parS*$_{pMT1}$ and part of *HNE_2270*, Kan^R	This study
pSRE10	pMCS-2 carrying *parS*$_{pMT1}$ and part of *HNE_3005*, Kan^R	This study
pSRE16	pZCHYN-8 carrying *parB*$_{pMT1}$, Tric^R	This study
pSW55	pYFPC-2 carrying *parB* (*HNE_3560*), Kan^R	This study

Supplementary Table 3. Construction of plasmids.

Plasmid	Description
pAJ34	a) amplification of *HNE_1677* with primer 146 (adding an *Nde*I site) and primer 147 (adding a *Kpn*I site)<br>b) ligation into pZVENC-2 cut with *Nde*I and *Kpn*I
pAJ36	a) amplification of part of *HNE_1854* with primer 173 (adding a *Kpn*I site) and primer 174 (adding a *Sac*I site)<br>b) ligation into pAR46 cut with *Sac*I and *Kpn*I
pAJ38	a) amplification of 547 bp 5' flanking region of *HNE_1677* with primer 177 (adding a *Hind*III site) and primer 178 (adding a *Kpn*I site)<br>b) amplification of 507 bp 3' flanking region of *HNE_1677* with primer 179 (adding a *Kpn*I site) and primer 180 (adding an *Eco*RI restriction site)<br>c) triple ligation into pNPTS138 cut with *Hind*III and *Eco*RI
pAJ40	a) amplification of *HNE_3560* with primer 148 (adding an *Nde*I site) and primer 186 (adding an *Eco*RI site)<br>b) ligation into pET21a+ cut with *Nde*I and *Eco*RI
pAJ45	a) isolation of the Rif^R cassette from pCCFPN-3 by restriction with *Nhe*I and *Sfi*I<br>b) ligation into pAR25 cut with *Nhe*I and *Sfi*I
pAJ46	a) amplification of 677 bp 5' flanking region of HNE_3561 with primer 216 (adding a *Hind*III restriction site) and primer 217 (adding a *Kpn*I restriction site)<br>b) amplification of 758 bp 3' flanking region of *HNE_3561* with primer 218 (adding a *Kpn*I restriction site) and primer 219 (adding an *Nhe*I restriction site)<br>c) triple ligation into pNPTS138 cut with *Hind*III and *Nhe*I
pAJ54	a) digestion of pSW55 (pYFPC-2+*HNE_3560*) with *Nde*I and *Eco*RI<br>b) ligation into pCERC-2 cut with *Nde*I and *Eco*RI
pAJ56	a) amplification of part of *HNE_1729* with primer 238 (adding *Kpn*I site) and primer 239 (adding *Nhe*I site)<br>b) ligation into pAR46 cut with *Nhe*I and *Kpn*I
pAJ57	a) amplification of part of *HNE_2644* with primer 240 (adding *Kpn*I site) and primer 241 (adding *Nhe*I site)<br>b) ligation into pAR46 cut with *Nhe*I and *Kpn*I
pAJ58	a) amplification of part of *HNE_0857* with primer 242 (adding a *KpnI* site) and primer 243 (adding a *Sac*I site)<br>c) ligation into pAR46 cut with *Sac*I and *Kpn*I
pAJ59	a) amplification of part of *HNE_0550* with primer 244 (adding *Kpn*I site) and primer 245 (adding *Sac*I site)<br>b) ligation into pAR46 cut with *Sac*I and *Kpn*I
pAJ60	a) amplification of part of *HNE_3540* with primer 246 (adding a *Kpn*I site) and primer 247 (adding a *Sac*I site)<br>b) ligation into pAR46 cut with *Sac*I and *Kpn*I
pAJ61	a) amplification of part of *HNE_0063* with primer 248 (adding a *Kpn*I site) and primer 249 (adding a *Sac*I site)<br>b) ligation into pAR46 cut with *Sac*I and *Kpn*I
pAJ62	a) isolation of insert from pAJ56 by restriction with *Hinc*II and *Nhe*I<br>b) ligation into pMCS-2 cut with *Hinc*II and *Nhe*I
pAJ63	a) isolation of insert from pAJ57 by restriction with *Sfi*I and *Nhe*I<br>b) ligation into pMCS-2 cut with *Sfi*I and *Nhe*I
pAJ64	a) isolation of insert from pAJ58 by restriction with *Hinc*II and *Nhe*I<br>b) ligation into pMCS-2 cut with *Hinc*II and *Nhe*I
pAJ65	a) isolation of insert from pAJ59 by restriction with *Sfi*I and *Nhe*I<br>b) ligation into pMCS-2 cut with *Sfi*I and *Nhe*I
pAJ66	a) isolation of insert from pAJ60 by restriction with *Sfi*I and *Nhe*I<br>b) ligation into pMCS-2 cut with *Sfi*I and *Nhe*I
pAJ67	a) isolation of insert from pAJ61 with *Hinc*II and *Nhe*I<br>b) ligation into pMCS-2 with *Hinc*II and *Nhe*I
pAJ70	a) isolation of Rif^R cassette from pCCFPN-3 by restriction with *Nhe*I and *Sfi*I<br>b) ligation into pAR51 cut with *Nhe*I and *Sfi*I
pAJ74	a) amplification of part of *parB* fused to *cerulean* with primer 299 (adding a *Hind*III site) and 257 (adding a *Kpn*I site) using pAJ54 as template<br>b) amplification of 838 bp of 3' flanking region of *parB* with primer 300 (adding a *Kpn*I site) and 301 (adding an *Nhe*I site)<br>c) triple ligation into pNPTS138 cut with *Hind*III and *Nhe*I
pAJ75	a) inverse PCR with primers 307 and 308 using pAR25 as template and subsequent *Dpn*I digest<br>b) isolation of mutated *parA* gene from the resulting plasmid with *Nde*I and *Kpn*I<br>c) ligation into pCVENC-2 cut with *Nde*I and *Kpn*I
pAJ78	a) isolation of *parA* gene from pAJ45 by restriction with *Nde*I and *Kpn*I<br>b) ligation into pCVENC-2 cut with *Nde*I and *Kpn*I
pAJ79	a) amplification of part of *HNE_1729* + downstream region with primer 311 (adding an *Eco*RI site) and primer 312 (adding an *Nhe*I site)<br>b) ligation into pKH3 cut with *Eco*RI and *Nhe*I

Supplementary Table 3. Construction of plasmids (continued).

Plasmid	Description
pAJ80	a) amplification of part of *HNE_3540* + downstream region with primer 313 (adding an *Eco*RI site) and primer 314 (adding an *Nhe*I site)<br>b) ligation into pKH3 cut with *Eco*RI and *Nhe*I
pAJ84	a) amplification of part of *dnaN* fused to *venus* with primer 345 (adding a *Hind*III site) and 315 (adding an *Nde*I site) using pSRE7 as template<br>b) amplification of the 3´ flanking region of *dnaN* with primers 343 (adding an *NdeI* site) and 344 (adding an *Eco*RI site)<br>c) triple ligation into pNPTS138 cut with *Hind*III and *Eco*RI
pSW55	a) amplification of *parB* (*HNE_3560*) without stop codon with primers oSW110 (adding an *Nde*I site) and oSW111 (adding an *Eco*RI site)<br>b) ligation into pYFPC-2 with *Nde*I and *Eco*RI
pAR25	a) amplification of part of HNE_2372 + upstream region with primers oSE1 (adding a *Hind*III site) and oSE4 (adding an *Nde*I site)<br>b) ligation into pXVENC-2 cut with *Hind*III and *Nde*I<br>c) digestion of the resulting plasmid with *Nde*I and and *Kpn*I<br>e) amplification of *parA* (*HNE_3561*) with primer oAR161 (adding an *Nde*I site) and primer oAR162 (adding a *KpnI* site)<br>f) ligation of the two fragments
pAR46	a) isolation of a $parS_{pMT1}$-containing fragment from pMS138 by restriction with *Hinc*II and *Nhe*I<br>b) ligation into pMCS-3 cut with *Hinc*II and *Nhe*I
pAR51	a) isolation of $parB_{pMT1}$ from pMS226 by restriction with *Kpn*I and *Nhe*I<br>b) ligation into pZCHYN-2 cut with *Kpn*I and *Nhe*I
pJR82	a) amplification of HNE_0032 with primers oMvT624 (adding an *Eco*RI site) and oMvT625 (adding an *Nhe*I site)<br>b) ligation into pKH3 cut with *Nhe*I and *Eco*RI
pKH3	a) amplification of *oriT* with primer oKH9 (adding an *Xba*I site) and oKH10 (adding an *NdeI* site) using pYFPC-2 as a template<br>b) ligation into pLAU43 cut with *Nde*I and *Xba*I
pKH4	a) amplification of part of *gyrB* and downstream region with primer oKH18 (adding an *Eco*RI site) and primer oKH38 (adding an *Nhe*I site)<br>b) ligation into pKH3 cut with *Eco*RI and *Nhe*I
pKH8	a) amplification of part of HNE_2372 and upstream region with primer SE1 (adding a *Hind*III site) and primer SE4 (adding an *Nde*I site)<br>b) ligation into pXCHYC-3 cut with HindIII and NdeI
pKH9	a) amplification of *lacI* with primers oKH22 (adding an *Nde*I site) and oKH23 (adding a *Sac*I site) using pLAU53 as a template<br>b) ligation into pKH8 cut with *Kpn*I and *Sac*I
pKH15	a) amplification of part of *hol*A and intergenic region with primer oKH3 (adding a *Bsr*GI site) and primer oKH49 (adding an *Nhe*I site)<br>b) ligation into pYFPC-2 cut with *Bsr*GI and *Nhe*I<br>c) restriction of resulting plasmid with *Mfe*I and *Sac*I<br>d) amplification of part of *parB* with primer oKH2 (adding a *SacI* site) and primer oKH48 (adding an *Mfe*I site)<br>e) ligation of the two fragments<br>f) isolation of the *parB-yfp-holA* fragment from the resulting plasmid with *Mfe*I and *Nhe*I<br>g) ligation into pNPTS138 cut with *Mfe*I and *Nhe*I
pSRE7	a) amplification of *dnaN* using primers oSRE14 (adding an *Nde*I site) and 318 (adding a *Kpn*I site)<br>b) ligation into pCVENN-2 cut with *Kpn*I and *Nde*I
pSRE8	a) amplification of part of *HNE_1299* with primer 328 (adding a *Kpn*I site) and primer 329 (adding a *Sac*I site)<br>b) ligation into pAJ67 cut with *Sac*I and *Kpn*I
pSRE9	a) amplification of part of *HNE_2270* with primer 326 (adding a *Kpn*I site) and primer 327 (adding a *Sac*I site)<br>b) ligation into pAJ67 cut with *Sac*I and *Kpn*I
pSRE10	a) amplification part of HNE_3005 using oligos 324 (adding a *Kpn*I site) and 325 (adding a *Sac*I site)<br>b) ligation into pAJ67 cut with *Kpn*I and *Sac*I
pSRE16	a) isolation of $Tric^R$ cassette from pCVENC-8 by restriction wih *Nhe*I and *Sfi*I<br>b) ligation into pAJ70 cut with *Nhe*I and *Sfi*I

Supplementary Table 4. Oligonucleotides used in this study.

ID	Name	Sequence (5' to 3')
**146**	HNE_1677 (PopZ) fw	atatCATatggccaacgaagcgcataaagaaccg
**147**	HNE_1677 (PopZ) rv fusion	atatGGTACCgcgcgccatgcgggcaatcc
**148**	ParB fw	atatCATatgagtgatccggcagaggacaatcgc
**173**	part HNE_1854 fw	atatGGTACCaagctctttcccgatcccccgccc
**174**	HNE_1854 rv	atGAGCTCtcagtccgggacatccggagacca
**177**	HNE_1677 F1fw	atatAAGCTTgatgccaaccagcgggacaccag
**178**	HNE_1677 F1rv	atatGGTACCgtcggttctttatgcgcttcgttgg
**179**	HNE_1677 F2fw	atatGGTACCgtacagcggattgcccgcatggc
**180**	HNE_1677 F2rv	atGAATTCgctgaccaacaattgcgccgccg
**186**	ParB rv fusion for pET	atGAATTCgattccgcagtccggcgcttagcg
**187**	HNE_1677 genom up	tccgccagaacaatgtgaccgtgacc
**188**	HNE_1677 genom down	cggatcccagttcacgaggcgcttg
**191**	pAJ36 integration check fw	tcaaggccgcacatctccaccgacg
**196**	Cy3 parS wt	ggtccaatgtttcacgtgaaacatcattggtttttccaatgatgtttcacgtgaaacattggacc
**197**	Cy3 parSmut	ggtccaatgcctcacgtgaaacatcattggtttttccaatgatgtttcacgtgaggcattggacc
**215**	parA genom up	gggtcaggccgggcggatcgaag
**216**	parA F1 fw	atatAAGCTTccgcgtccggaaagctgggtgac
**217**	parA F1 rv	atatGGTACCttgattcaccacggcaaatattc
**218**	parA F2 fw	atatGGTACCgaagtgttgcagcgtgaaagggc
**219**	parA F2 rv	atataGCTAGCggcttcgacctcccggacgctga
**220**	parA genom down	agcggcggcagacttcatcgagc
**238**	part HNE_1729 fw	atatGGTACCgcccggatcgctgatgagctgctc
**239**	HNE_1729 rv	atataGCTAGCtcatggagctgcaaatctcatcacttctgc
**240**	part of HNE_2644	atatGGTACCacggtcaccaacggcatcatctccg
**241**	HNE_2644 rv	atataGCTAGCtcaggtgcgcactgtgcctcgc
**242**	part of HNE_0857 fw	atatGGTACCaaggacatcggcgcggtgatctgc
**243**	HNE_0857 rv	atGAGCTCtcagcccacccgctccaccacaac
**244**	part of HNE_0550 fw	atatGGTACCgcccgcccctaaagcgcctcc
**245**	HNE_0550 rv	atGAGCTCtcatcgagcggggcggggagggc
**246**	part of HNE_3540 fw	atatGGTACCgggcgctggatgaaaaccaaggac
**247**	HNE_3540 rv	atGAGCTCtcaccccatcggatagagaatttcccg
**248**	part of HNE_0063 fw	atatGGTACCggaccgcgaatggatggatgcct
**249**	HNE_0063 rv	atGAGCTCtcacatttctttcgggggcttcaggc
**250**	int check pAJ56	cggcgcctagaagctcggacgatg
**251**	int check pAJ57	gctgctcgcggctgccttcctcg
**252**	int check pAJ58	accccgatatcatgaccccgcgc
**253**	int check pAJ59	gcccttcacctcccatcgctttgc
**254**	int check pAJ60	ggatcaactttttcgacacggccg
**255**	int check pAJ61	gccgcaatcagcaccaagtcgcc
**257**	cerulean rv F2	atatGGTACCttacttgtacagctcgtccatgccg
**299**	parB fw HindIII	atatAAGCTTatgagtgatccggcagaggacaatcgc
**300**	part of holA fw F3	atatGGTACCtaatccgttaacgaacgttttcag
**301**	part of holA rv F4	atataGCTAGCggcggcccgatccggaagtctgg
**306**	parB genom down 3	gatgctgcacaaggggcgcgcgg
**307**	parA K18R mut fw	caaaagggcggggtcggaagaaccacgacctcgatcaatc
**308**	parA K18R mut rv	gattgatcgaggtcgtggttcttccgaccccgcccttttg
**311**	part of HNE_1729 FROS fw	atGAATTCttttgacgatgccaacaaaatgcc
**312**	inter HNE_1729 FROS rv	atataGCTAGCagtggtacgtgccccgcctgctac
**313**	part of HNE_3540 FROS fw	atGAATTCcgggcgctggatgaaaaccaaggac
**314**	inter HNE_3540 FROS rv	atataGCTAGCgccctcccttggctcccggtctag
**315**	venus rv F2 (NdeI)	atatCATATGttacttgtacagctcgtccatgccg
**318**	dnaN no stop rv	atatGGTACCcacccgcagcggcatcacgacaa
**320**	downstr HNE_1729 for seq	tagcgaactgactggcgcgcgcg
**321**	downstr HNE_3540 for seq	gcaaggcgaggtctgcggcgagg
**322**	int. Check pAJ79 (FROS ter)	tcgggctcttcggaacggcatcg
**323**	int. Check pAJ80 (FROS ori)	cggcggtggcagcgatgatgatg
**324**	part of HNE_3005 fw	atatGGTACCtgcgttgccaagatcgagaccgc

Supplementary Table 4. Oligonucleotides used in this study (continued).

ID	Name	Sequence (5' to 3')
**325**	HNE_3005 rv	atGAGCTCtcagtcttccagctggcgcccctc
**326**	HNE_2270 +upstr. fw	atatGGTACCaagaaggggcgcgcggagtggac
**327**	HNE_2270 rv	atGAGCTCtcaaaccttcttcacaaactccg
**328**	part of HNE_1299	atatGGTACCgcgcccttctcggcctgcaaacc
**329**	HNE_1299 rv	atGAGCTCtcaggtgcggaaggagcggcgg
**333**	int. Check pAJ80 (FROS ori)-2	ccatgccggcaaacgcgaagtcc
**343**	dnaN down fw F3	atatCATATGgccgtgcagacgggccgccgcatc
**344**	dnaN down rv F4	atGAATTCcgcgtcgatggcgatctgcagag
**345**	dnaN fw F1	atatAAGCTTatgaaactgacgatcgaacgcggagac
**oKH3**	holA_for	cTGTACAagtaataatccgttaacgaacg
**oKH49**	holA_rev	tcgaGCTAGCcgaagctgtctcgct
**oKH2**	parB_rev	gGAGCTCcttccgcagtccggcg
**oKH48**	parB_for	cCAATTGacaaacgagatcgccatc
**oKH9**	*oriT*_rev	tagtTCTAGAgcggccgtcgacgga
**oKH10**	*oriT*_for	agatCATATGacacaacgtggcttt
**oKH22**	*lacI*_for	ttcCATATGgtggtgaatgtgaaac
**oKH23**	*lacI*_rev	aGAGCTCgaccgcccagctgcatta
**oKH18**	*gyrB_for*	tGAATTCgattgcgagaagatcccg
**oKH38**	*gyrB_rev*	gGCTAGCcagaactcctacaaggtc
**oAR161**	HNE_3561 for	cgtcCATATGactagacccagaatatttgc
**oAR162**	HNE_3561 rev	aGGTACCtgccgccctcgccct
**oMvT624**	FROS2_p1	atatGAATTCcatctcggtcagtgccagccacattg
**oMvT625**	FROS2_p2	atatgctagCcggcgctcctgctttgcctgtc
**oSE1**	HNE_2372-1 forw	atAAGCTTgagagaccgcgtcaatgaccagcg
**oSE4**	HNE_2372-4 rev	aattaaCATATGccgcgcccgacaggcgcattcg
**oSW110**	HNE_3560-for	ttaaCATATGagtgatccggcagaggacaatcgc
**oSW111**	HNE_3560-rev	taGAATTCgattccgcagtccggcgcttagcggtc
**oSRE14**	dnan_Cter_fw	atatCATatgaaactgacgatcgaacgcggagac

Supplementary references

1. Bowman GR, Perez AM, Ptacin JL, Ighodaro E, Folta-Stogniew E, Comolli LR, Shapiro L (2013) Oligomerization and higher-order assembly contribute to sub-cellular localization of a bacterial scaffold. *Mol Microbiol* **90:** 776-795.

2. Laloux G, Jacobs-Wagner C (2013) Spatiotemporal control of PopZ localization through cell cycle–coupled multimerization. *J Cell Biol* **201:** 827-841.

3. Leifson E (1964) *Hyphomicrobium neptunium* sp. n. *Antonie van Leeuwenhoek* **30:** 249-256.

4. Jung A, Eisheuer S, Cserti E, Leicht O, Strobel W, Möll A, Schlimpert S, Kühn J, Thanbichler M (2015). Molecular toolbox for genetic manipulation of the stalked budding bacterium *Hyphomonas neptunium*. *Appl Environ Microbiol* **81:** 736-744.

5. Thanbichler M, Iniesta AA, Shapiro L (2007) A comprehensive set of plasmids for vanillate- and xylose-inducible gene expression in *Caulobacter crescentus*. *Nucleic Acids Res* **35:** e137.

6. Lau IF, Filipe SR, Søballe B, Økstad OA, Barre FX, Sherratt DJ (2004) Spatial and temporal organization of replicating *Escherichia coli* chromosomes. *Mol Microbiol* **49:** 731-743.

7. Schwartz MA, Shapiro L (2011) An SMC ATPase mutant disrupts chromosome segregation in *Caulobacter*. *Mol Microbiol* **82:** 1359-1374.
