# Supplementary figures and images for: Two-step chromosome segregation in the stalked budding bacterium Hyphomonas neptunium
Source: Nat Commun. 2019 Jul 23;10:3290. doi: 10.1038/s41467-019-11242-5 (PMC6650430; doi:10.1038/s41467-019-11242-5)

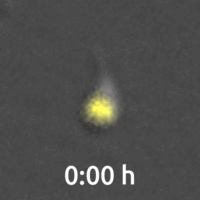

Supplement: Supplementary file 3 — Supplementary movie 1 [file 41467_2019_11242_MOESM3_ESM.gif]
